# Posttranscriptional Regulation of *tnaA* by Protein-RNA Interaction Mediated by Ribosomal Protein L4 in Escherichia coli

**DOI:** 10.1128/JB.00799-19

**Published:** 2020-04-27

**Authors:** Dharam Singh, Oleg N. Murashko, Sue Lin-Chao

**Affiliations:** a Institute of Molecular Biology, Academia Sinica, Taipei, Taiwan; Ohio State University

**Keywords:** extraribosomal function, gene regulation, mRNA stability, posttranscriptional regulation, protein-RNA interactions

## Abstract

Escherichia coli ribosomal protein (r-protein) L4 has extraribosomal biological functions. Previously, we described L4 as inhibiting RNase E activity through protein-protein interactions. Here, we report that from stabilized transcripts regulated by L4-RNase E, mRNA levels of *tnaA* (encoding tryptophanase from the *tnaCAB* operon) increased upon ectopic L4 expression, whereas TnaA protein levels decreased. However, at nonpermissive temperatures (to inactivate RNase E), *tnaA* mRNA and protein levels both increased in an *rne* temperature-sensitive [*rne*(Ts)] mutant strain. Thus, L4 protein fine-tunes TnaA protein levels independently of its inhibition of RNase E. We demonstrate that ectopically expressed L4 binds with transcribed spacer RNA between *tnaC* and *tnaA* and downregulates TnaA translation. We found that deletion of the 5′ or 3′ half of the spacer compared to the wild type resulted in a similar reduction in TnaA translation in the presence of L4. *In vitro* binding of L4 to the *tnaC-tnaA* transcribed spacer RNA results in changes to its secondary structure. We reveal that during early stationary-phase bacterial growth, steady-state levels of *tnaA* mRNA increased but TnaA protein levels decreased. We further confirm that endogenous L4 binds to *tnaC-tnaA* transcribed spacer RNA in cells at early stationary phase. Our results reveal the novel function of L4 in fine-tuning TnaA protein levels during cell growth and demonstrate that r-protein L4 acts as a translation regulator outside the ribosome and its own operon.

**IMPORTANCE** Some ribosomal proteins have extraribosomal functions in addition to ribosome translation function. The extraribosomal functions of several r-proteins control operon expression by binding to own-operon transcripts. Previously, we discovered a posttranscriptional, RNase E-dependent regulatory role for r-protein L4 in the stabilization of stress-responsive transcripts. Here, we found an additional extraribosomal function for L4 in regulating the *tna* operon by L4-intergenic spacer mRNA interactions. L4 binds to the transcribed spacer RNA between *tnaC* and *tnaA* and alters the structural conformation of the spacer RNA, thereby reducing the translation of TnaA. Our study establishes a previously unknown L4-mediated mechanism for regulating gene expression, suggesting that bacterial cells have multiple strategies for controlling levels of tryptophanase in response to varied cell growth conditions.

## Abstract (precis)

Some ribosomal proteins have extraribosomal functions in addition to ribosome translation function. The extraribosomal functions of several r-proteins control operon expression by binding to own-operon transcripts. Previously, we discovered a posttranscriptional, RNase E-dependent regulatory role for r-protein L4 in the stabilization of stress-responsive transcripts. Here, we found an additional extraribosomal function for L4 in regulating the *tna* operon by L4-intergenic spacer mRNA interactions. L4 binds to the transcribed spacer RNA between *tnaC* and *tnaA* and alters the structural conformation of the spacer RNA, thereby reducing the translation of TnaA. Our study establishes a previously unknown L4-mediated mechanism for regulating gene expression, suggesting that bacterial cells have multiple strategies for controlling levels of tryptophanase in response to varied cell growth conditions.

## INTRODUCTION

Some ribosomal proteins (r-proteins) in eukaryotes and prokaryotes exhibit extraribosomal functions (1–4). Several r-proteins autogenously control the expression of their operons by binding to their own mRNAs. Additional extraribosomal functions arise from interactions with nonribosomal components, such as specific proteins or RNA molecules. Although the molecular mechanisms of autogenous r-protein synthesis control have been studied extensively (5, 6), other extraribosomal functions are less understood.

R-protein L4, a member of the S10 r-protein operon, autogenously controls the synthesis of its own ribosomal proteins in E. coli (7, 8). As is the case for many other r-proteins, feedback regulation by L4 operates either by inhibiting translation of its polycistronic mRNA (9) or inhibiting transcription of its S10 operon by binding to independent but overlapping determinants within the 5′ untranslated region (5′-UTR) (6, 10, 11). Apart from its RNA-binding sites, L4 hosts multifunctional domains for interactions with other proteins (12, 13). Indeed, L4 was found to interact with 64 proteins in E. coli (14). We previously reported that L4 interacts with the C-terminal region of RNase E to regulate its activity, leading to the stabilization of specific stress-responsive mRNAs critical for cell survival in E. coli (4). In eukaryotes, L4 has been shown to functionally interact with the RNA helicase II/Guα in mammalian cells (15) and with calnexin in Madin-Darby canine kidney cells (16). In plants, plastid L4 might play a role in plastid transcriptional regulation (17). Despite the specific protein-RNA interactions demonstrated for many r-proteins in ribosomes (18), their RNA-based extraribosomal regulatory mechanisms are still being discovered.

RNase E-dependent and specific RNA targets of L4 have been reported, and it was revealed that the targets most affected were transcripts from the tryptophanase (*tnaCAB*) operon (4). This operon consists of a transcribed leader peptide, TnaC, followed by two structural genes, i.e., tryptophanase (TnaA, an enzyme involved in amino acid degradation) and tryptophan-specific permease (TnaB) (19). Expression of the *tnaCAB* operon is controlled by catabolite repression (20) and tryptophan-specific induction (21). Here, we show that steady-state levels of mRNA transcripts from *tnaCAB* increased upon ectopic expression of L4, whereas levels of TnaA protein decreased. We found that L4 specifically binds to the region upstream of the *tnaA* coding sequence, potentially affecting the structural conformation of the RNA region upstream of TnaA to reduce its translation. L4 binding does not affect translation of *tnaC*, which lies upstream of *tnaA*. We discuss the biological significance of the molecular mechanism by which L4 inhibits *tnaA* translation.

## RESULTS

### L4 alters *tnaA* mRNA and protein output in the wild type but not an RNase E temperature-sensitive strain.

**FIG 1:**
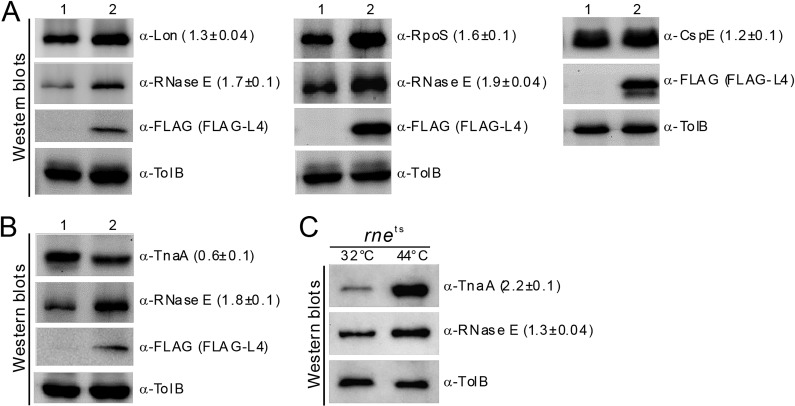
Western blot analysis of proteins from transcripts regulated by L4 in an RNase E-dependent manner. (A and B) Bacterial cultures were collected from the wild-type E. coli N3433 strain carrying plasmid pPW500flag (lane 1) or plasmid pPWflagL4 encoding L4 (lane 2) after induction with IPTG (0.5 mM) for 30 min. The Western blots were analyzed for Lon (87 kDa), RpoS (38 kDa), CspE (8 kDa), and TnaA (53 kDa) using specific antibodies by loading samples on 10% SDS-PAGE (except 15% SDS-PAGE for CspE). The values in parentheses indicate fold changes in protein levels, measured from at least two independent biological repeats. The effect of FLAG-L4 induction on the increase in RNase E protein levels was verified using antibodies against FLAG peptide (α-FLAG) and RNase E (α-RNase E). TolB was used as a loading control. (C) Western blot detection of TnaA protein in a temperature-sensitive mutant *rne* N3431 strain at permissive (32°C) and nonpermissive (44°C) temperatures using TnaA-specific antibody (α-TnaA) by loading samples on 10% SDS-PAGE. Western blots for RNase E and TolB are also shown. Results are expressed as means ± SEM.

In a previous study (4), we found that a subset of transcripts showed differentially increased mRNA stability/abundance upon ectopic L4 expression. Therefore, we first examined whether mRNA stabilization/increased transcript abundance is correlated with the levels of protein synthesis by using Western blotting to assess final gene products. We found ectopic L4 induction increased protein levels of RNaseE (∼1.8-fold) and RpoS (∼1.6-fold) and very mildly affected Lon (∼1.3-fold) and CspE (∼1.2-fold) proteins in E. coli wild-type strain N3433 compared to cells containing a control plasmid (Fig. 1A). Unexpectedly, ectopic L4 expression resulted in decreased levels of endogenous tryptophanase (TnaA) (Fig. 1B), though steady-state levels of *tnaA* mRNA increased. The reduced levels of TnaA upon L4 expression could be the result of rapid TnaA degradation. To investigate this possibility, we arrested translation with chloramphenicol and then compared the protein stability of TnaA upon L4 expression with that of control plasmid (i.e., lacking inducible L4). Our data indicate that ectopic L4 expression does not alter TnaA protein half-life (>128 min) and does not promote faster TnaA degradation (see Fig. S1 in the supplemental material). Thus, we observed a decrease in TnaA protein levels despite an increase in *tnaA* transcript abundance (4), suggesting the existence of a novel regulatory mechanism that is independent of the L4-RNase E regulatory pathway. Furthermore, we analyzed expression levels of TnaA protein in an *rne* temperature-sensitive [*rne*(Ts)] mutant strain. RNase E inactivation in the *rne*(Ts) strain at a nonpermissive temperature resulted in an expected increase in TnaA protein levels (TnaA levels increased ∼2.2-fold after shifting to 44°C) (Fig. 1C), suggesting that the *tna* transcript is also regulated by RNase E in a manner independent of L4-specific regulation of TnaA protein levels. These results indicate that expression of the TnaA protein of the *tnaCAB* operon is reduced upon ectopic expression of L4 in wild-type cells.

**FIG 2:**
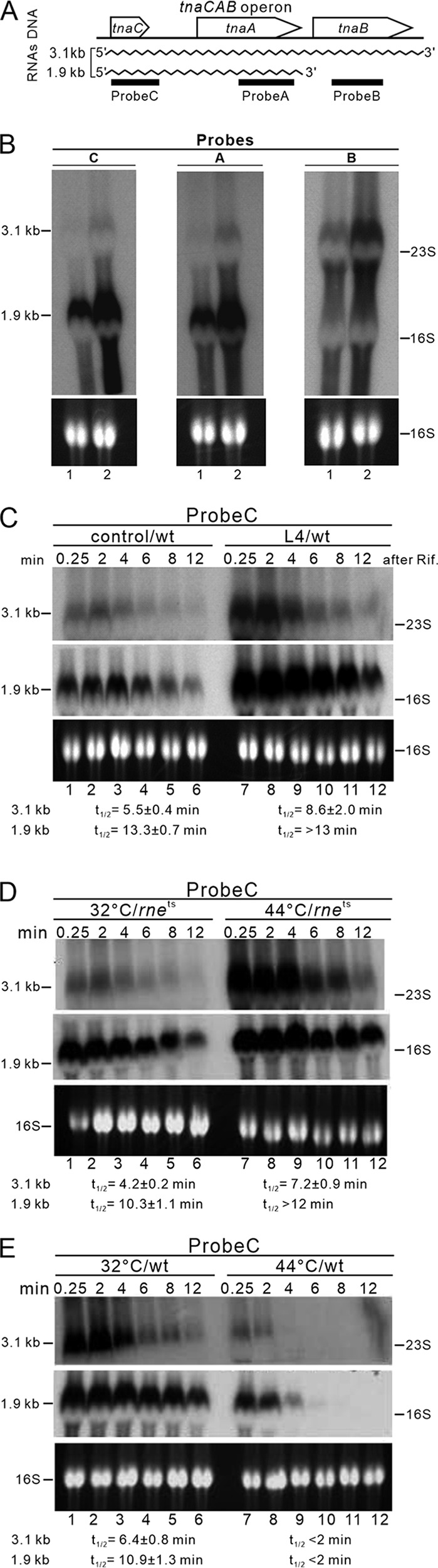
Northern blot analysis of *tna* transcripts in the presence or absence of ectopically expressed L4 in an RNase E wild-type E. coli strain (N3433) or an RNase E temperature-sensitive mutant [*rne*(Ts)] strain (N3431) grown at 32°C or 44°C. (A) The positions of the transcription unit and individual probes of the *tnaCAB* operon are shown. The antisense RNA probes A, B and C were synthesized using T7 RNA polymerase and used for Northern blot detection of their respective transcripts. The wavy lines show the extent of the full-length (3.1 kb) and intermediate (1.9 kb) *tna* transcripts. (B) Northern blot analysis of steady-state *tna* transcripts hybridized with probes A, B, and C as indicated for each blot. After induction for 30 min with IPTG (0.5 mM), steady-state total RNA was isolated from the N3433 strain (WT) carrying plasmid pPW500flag (lane 1) or plasmid pPWflagL4 (lane 2). Note that levels of the 3.1-kb transcript without L4 were very low compared to when L4 was ectopically expressed. Therefore, we used overexposed film to quantify the 3.1 kb signal. (C to E) Northern blot analysis determining the half-life of *tna* mRNA in the N3433 strain carrying pPWflag500 or pPWflagL4 plasmids or in N3431 [*rne*(Ts)] and N3433 (WT) strains grown at 32°C or 44°C. After a 30-min induction of L4 with IPTG (0.5 mM) or after shifting to a nonpermissive temperature (44°C) for 30 min, total RNA was isolated in each strain at the indicated time after stopping RNA transcription with rifampin. Equal amounts of total RNA were analyzed by Northern blotting using radiolabeled RNA probes specific for *tnaC* mRNA. After shifting to a nonpermissive temperature (44°C), only the 3.1-kb and 1.9-kb RNA species were detected in the wild-type RNase E strain until the second time point, 2 min after rifampin treatment. Both RNA species have a half-life of less than 2 min. Half-life curves representing two biological repeats of *tna* transcripts are shown in Fig. S2. RNA half-lives were determined by nonlinear regression curve fitting (one phase decay) using GraphPad Prism. Results are expressed as means ± SEM.

To address this, we investigated the steady-state levels of *tnaC* and *tnaA* transcripts in the wild-type (WT) N3433 strain upon ectopic L4 expression. We used three specific probes (Fig. 2A) derived from the *tna* operon (probe A [*tnaA*], probe B [*tnaB*], and probe C [*tnaC*]) and detected a sharp increase in the levels of 3.1-kb (∼3- to 5-fold) and 1.9-kb (∼3-fold) transcripts relative to basal expression with the control plasmid (Fig. 2B, compare lanes 1 and 2). The 3.1-kb transcript was detected by all three probes, whereas only probes A and C strongly detected the 1.9-kb RNA transcript, indicating that the 1.9-kb RNA fragment is at the 5′ end of the *tna* transcript and encodes TnaC and TnaA but not TnaB. Next, we determined the impact of ectopic L4 expression on transcript stability. We found that the half-lives of the 3.1-kb and 1.9-kb RNA species were more stable upon L4 expression (half-lives increased from ∼6 to ∼9 min and ∼13 to >13 min, respectively) (Fig. 2C, Fig. S2A, and Table S1). These data indicate that the L4-RNase E pathway can also regulate the stability of these RNA species.

Next, we characterized *tna* mRNA stability by rifampin treatment and determined the rate at which *tna* transcript disappeared over time by Northern blotting analysis. We used isogenic strains containing or lacking RNase E activity (i.e., under nonpermissive temperatures). We found that after shifting the temperature-sensitive N3431 strain, but not the WT N3433 strain, to a nonpermissive temperature (44°C), the stability of *tna* transcripts (the 3.1-kb and 1.9-kb RNA species) increased compared to the status of the strain at 32°C (Fig. 2D, compare lanes 7 to 12 with lanes 1 to 6; Fig. S2B; Table S1). In contrast, the 3.1-kb and 1.9-kb RNA species were quite unstable in the N3433 wild-type strain at 44°C (Fig. 2E, compare lanes 7 to 12 with lanes 1 to 6; Fig. S2C; Table S1). These data show that the 3.1-kb and 1.9-kb RNA species are stabilized in the RNase E temperature-sensitive strain under a nonpermissive temperature (44°C), indicating that degradation of the *tna* transcripts (3.1 and 1.9 kb) is dependent on RNase E enzymatic activity. Thus, the half-life of *tna* mRNA is longer when the activity of RNase E is reduced/depleted either by L4 inhibition or by temperature inactivation.

### L4 does not affect expression of *tna* transcriptional fusion constructs but downregulates translational fusion of the *tnaC-tnaA* transcribed spacer mRNA.

We found that L4 expression increased steady-state levels of *tna* transcripts while simultaneously reducing TnaA protein levels. Whereas the increased transcript levels might be due to increased promoter activity and mRNA stabilization (Fig. 2), the decreased TnaA protein levels could result from L4-mediated translational repression. We investigated this mechanism by creating transcriptional and translational *tnaCAB* fusion operons with *lacZ* to determine β-galactosidase activity *in vivo* upon L4 ectopic expression.

**FIG 3:**
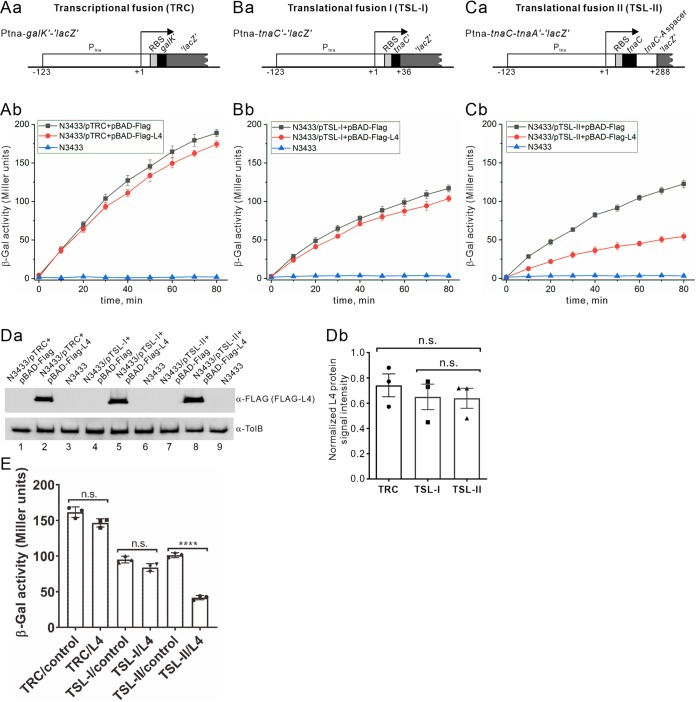
Determination of β-galactosidase activity in *tna* transcriptional and translational fusion constructs in the presence or absence of ectopically expressed L4. (A to C) Schematic representations of the plasmids used for transcriptional (Aa) or translational (Ba and Ca) fusion studies and β-galactosidase assays to study the effect of L4 on transcription (Ab) or translation (Bb and Cb) of the β-galactosidase fusion constructs. We used pMU575 (22) for transcriptional fusion of the *tna* promoter (−123 to +1 of the Ptna-*galK’-’lacZ*’ transcriptional fusion construct or TRC in panel Aa). Plasmid pMU2386 derived from pMU525 (23) was used for the construction of the *tna* translational fusion construct Ptna-*tnaC’-’lacZ*’, which lacks the *tnaC-tnaA* spacer RNA (TSL-I in panel Ba), or Ptna-*tnaC-tnaA’-’lacZ*’ with the *tnaC-tnaA* spacer RNA (TSL-II in panel Ca). Derived *tna* operon coordinators of their respective translational fusion constructs are also indicated. RBS, ribosomal binding site. Transcriptional (pTRC) or translational (pTSL-1 or pTSL-II) fusion vectors were cotransformed with either pBAD-Flag-L4 or pBAD-Flag in N3433 cells. Plasmid-free N3433 was used as a control for background β-galactosidase activity. Flag-L4 was induced with 0.2% arabinose for 30 min (cultured at 32°C, 180 rpm). The β-galactosidase assay was performed according to the method of Miller (53) and is described in Materials and Methods. β-Galactosidase activity was measured every 10 min for 80 min after adding the β-galactosidase substrate *o*-nitrophenyl-β-d-galactopyranoside (ONPG). N3433 with the plasmids for each assay are indicated. Error bars indicate ±SEM. (D) Western blot (a) and quantification (b) of ectopically expressed Flag-L4 protein levels in the above strains. TolB was used for normalization. *n* = 3; mean ± SEM; n.s., no significance after one-way ANOVA and Tukey’s *post hoc* test. (E) Quantification of β-galactosidase activity for transcriptional and translational fusion constructs. For quantification, data were taken at the 60-min time point after adding ONPG. Background β-galactosidase activity in N3433 was subtracted from each assay. *n* = 3; mean ± SEM; ****, *P* < 0.0001; n.s., no significance after one-way ANOVA and Tukey’s *post hoc* test.

We used a single copy number pMU575 vector for transcriptional fusion (22) of the *tna* promoter (nucleotides [nt] −123 to +1, Ptna-*galK′-’lacZ*’). For translational fusion of the *tnaCAB* operon with *lacZ*, a pMU2386 vector derived from pMU525 that lacks an appropriate translation start signal (23) was fused with the *tna* operon (nt −123 to +36, Ptna-*tnaC’-’lacZ*’) (Fig. 3A and B). We determined β-galactosidase activity in cells harboring the Ptna-*galK′-’lacZ*’ transcriptional fusion construct and found no significant differences in the presence or absence of ectopic L4 expression (Fig. 3A). Furthermore, there was no difference in β-galactosidase activity for bacterial cells carrying the translational fusion *lacZ* plasmid lacking the *tnaC-tnaA* spacer region, with or without ectopic L4 expression (Fig. 3B). This outcome suggests that regulatory elements downstream of the promoter in the *tnaC-tnaA* transcribed spacer region are responsible for L4-mediated downregulation of TnaA translation. We constructed an additional translational fusion plasmid, Ptna-*tnaC-tnaA’-’lacZ*’ (Fig. 3Ca), which produces mRNA transcripts encoding TnaC and a TnaA’–β-galactosidase fusion protein. The transcript also contains a spacer between *tnaC* and *tnaA*. We determined and compared β-galactosidase activities in cells harboring this translational fusion plasmid in the presence or absence of ectopic L4 expression. The results (Fig. 3Cb) show that upon ectopic L4 expression in cells harboring the Ptna-*tnaC-tnaA’-’lacZ*’ translational fusion construct β-galactosidase activity was significantly reduced by ∼2.3-fold (Fig. 3Cb and quantitative data in Fig. 3E, compare control versus L4). In all cell cultures used for this study, levels of ectopically expressed L4 (tagged with an N-terminal Flag peptide) are similar (Fig. 3D). Our results indicate that ectopically expressed L4 can downregulate the Ptna-*tnaC-tnaA’-’lacZ*’ but not Ptna-*tnaC’-’lacZ*’ translational fusion construct and that L4 has no significant effect on the Ptna-*galK’-’lacZ*’ transcriptional fusion construct, together demonstrating that L4 regulation of TnaA synthesis requires the *tnaCA* spacer region.

### L4 binds specifically to the *tnaC-tnaA* transcribed spacer *in vitro* and *in vivo*.

**FIG 4:**
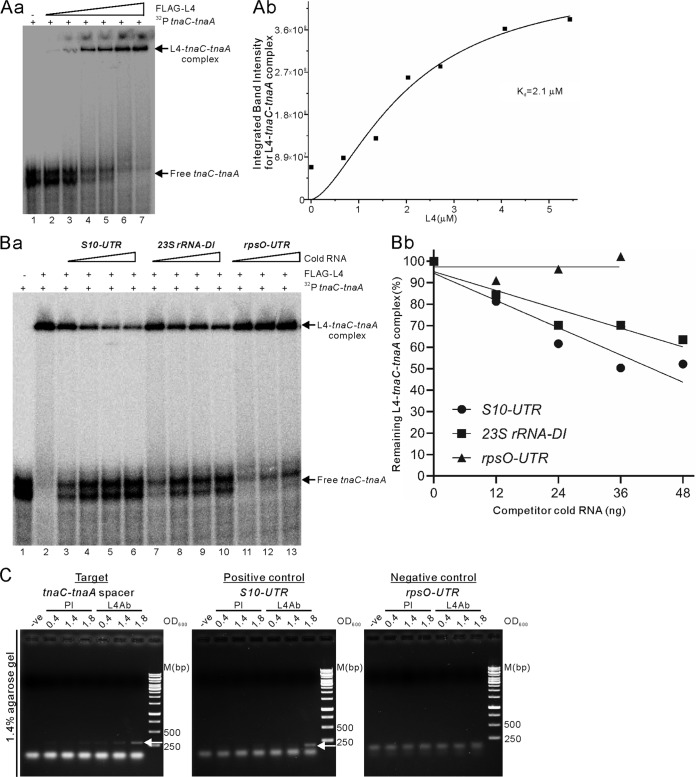
L4 specifically binds to the transcribed spacer between *tnaC* and *tnaA in vitro* and *in vivo*. (A) Mobility gel shift assay of L4 and *tnaC-tnaA* spacer RNA interactions. *In vitro* transcription of [α-^32^P]UTP-labeled *tnaC-tnaA* spacer RNA (220 nt, 30,000 cpm, ∼2 ng) and mobility gel shift assays were performed as described in Materials and Methods. (Aa) Mobility gel shift assay in the absence (lane 1) or increasing amounts (lanes 2 to 7; 0.7, 1.4, 2.1, 2.7, 4.1, and 5.5 μM, respectively) of FLAG-L4, mixed at 30°C for 20 min and separated in a nondenaturing 5% polyacrylamide gel. The positions of bound and free RNA species are indicated. (Ab) Binding affinity of the L4-*tnaC-tnaA* complex. L4 binds to *tnaC-tnaA* spacer with a high affinity (*K_d_* = 2.1 μM). (Ba) Altered mobility of the *tnaC-tnaA* spacer RNA was determined in a competition assay using L4 binding to an excess of cold RNA competitors. Competitive binding of L4 to specific targets S10-UTR RNA (lanes 3 to 6) or 23S rRNA-DI (lanes 7 to 10) or to the nonbinding target *rpsO*-UTR RNA (lanes 11 to 13). The cold excess of L4-binding RNA was 12, 24, 36, and 48 ng, and for the nonbinding RNA it was 12, 24, and 36 ng, while maintaining a constant amount of radiolabeled *tnaC-tnaA* spacer RNA (30,000 cpm; ∼2 ng). The competitor RNAs were in excess of ∼6- to 24-fold (for L4-binding targets) or ∼6- to 18-fold (for the nonbinding target) over that of the radiolabeled *tnaC-tnaA* spacer RNA. (Bb) Graph representing the percentage of remaining L4-*tnaC-tnaA* complexes competing with excess (as indicated by the *x* axis) cold individual competitor RNA. (C) Polyclonal L4 antibody coimmunoprecipitation (co-IP) assay confirming complex formation of the *tnaC-tnaA* spacer RNA with L4 in E. coli N3433 cells. Cell cultures for L4 co-IP using L4 polyclonal antibody were collected at different cell growth stages (OD_600_ = 0.4, 1.4, and 1.8), the RNA was isolated, and then reverse transcription and PCR were performed as described in the Materials and Methods using specific primers for the *tnaC-tnaA* spacer region, S10-UTR (as an L4-binding positive control), and *rpsO*-UTR (a nonbinding control). The expected sizes of the PCR products for the *tnaC-tnaA* spacer (261 bp [lanes 2 to 3 of L4Ab in the left panel]) or S10-UTR (177 bp [lane 3 of L4Ab in the middle panel]) are shown by arrows, or there was no amplified product for *rpsO*-UTR (right panel). Each product was resolved on a 1.4% agarose gel and visualized after staining with ethidium bromide dye. The same experiment was performed on each RNA species but with preimmune (PI) serum (lanes 1 to 3), which resulted in no detectable signal. The protein A-Sepharose beads incubated with total lysate but without L4 antibody also showed no detectable signals (shown as −ve). The DNA size marker is indicated [M(bp)].

L4 has multiple functional domains and is known to repress both transcription and translation of its S10 operon by binding to its 5′-UTR and 23S rRNA (7, 24, 25). Accordingly, it can potentially interact with diverse specific targets. Using purified FLAG-L4 protein and an [α-^32^P]UTP-labeled *tnaC-tnaA* spacer RNA fragment (∼220 nt) in an electrophoretic mobility gel shift assay, we observed a slower-migrating band of stable L4-*tnaC-tnaA* transcribed spacer RNA complexes (Fig. 4Aa, lanes 2 to 7). L4 binds to the *tnaC-tnaA* spacer with an affinity of dissociation constant (*K_d_*) = 2.1 μM (Fig. 4Ab). To ascertain if L4 specifically binds the *tnaC-tnaA* transcribed spacer RNA, we used the known L4-specific RNA targets S10-UTR (10) and 23S rRNA-DI (11) as positive competitors. Stelzl et al. (26) found that addition of an *rpsO*-UTR 218-nt RNA, which contains the interaction site for r-protein S15 (RpsO) that regulates translation of the *rpsO* operon, did not affect complex formation between the known L4-specific RNA target S10-UTR and r-protein L4 (RplD). Therefore, in this study, we used the *rpsO*-UTR as a nonspecific competitor and compared their competitive binding with that of L4 to the α-^32^P-labeled *tnaC-tnaA* spacer RNA complex. In this competition experiment, increasing doses of cold S10-UTR RNA or 23S rRNA-DI successfully competed with L4 for binding of labeled *tnaC-tnaA* spacer RNA, leading to an increase in free α-^32^P-labeled *tnaC-tnaA* spacer RNA fragments released from the bound complex (Fig. 4Ba, lanes 3 to 6 versus lane 2 for S10-UTR RNA and lanes 7 to 10 versus lane 2 for 23S rRNA-DI RNA). In contrast, addition of an increased concentration of cold nonspecific competitor *rpsO*-UTR did not significantly alter amounts of the labeled L4-*tnaC-tnaA* spacer RNA complex (Fig. 4Ba, lanes 11 to 13). Therefore, the nonspecific competitor (*rpsO*-UTR) did not successfully compete with the *tnaC-tnaA* spacer RNA for L4 binding. This competitive binding assay also shows that S10-UTR and 23S rRNA-DI are strong competitors in terms of their affinity for L4 (Fig. 4Bb). Our results experimentally demonstrate that L4 specifically binds within the *tnaC-tnaA* transcribed spacer region.

We performed an L4 coimmunoprecipitation (co-IP) assay (using specific antibodies against L4 protein) on N3433 cells at different growth stages and then performed reverse transcriptase PCR (RT-PCR) assays to demonstrate binding of L4 to the *tnaC-tnaA* spacer in E. coli cells. Using specific forward and reverse primers to detect the *tnaC-tnaA* spacer, we observed the expected 261-bp fragment in the L4 pulldown portion, and amounts of the 261-bp fragment were significantly increased in cells in stationary-phase growth (at optical density at 600 nm [OD_600_] values of 0.4, 1.4, and 1.8, where the levels were highest at 1.8) (Fig. 4C, left panel). As a positive control, we generated the expected 177-bp fragment (Fig. 4C, middle panel) in the co-IP assay using specific forward and reverse primers to detect S10-UTR. In contrast, specific forward and reverse primers for the *rpsO*-UTR region did not generate any amplified fragments in the L4 pulldown portion upon RT-PCR (Fig. 4C, right panel), which is consistent with lack of interaction between L4 and *rpsO*-UTR. Collectively, our results suggest a possible mechanism by which L4 regulates TnaA translation by specifically binding to the *tnaC-tnaA* spacer RNA region.

### L4 differentially downregulates *tnaA* translation rather than *tnaC* translation in a cell-free transcription/translation system.

**FIG 5:**
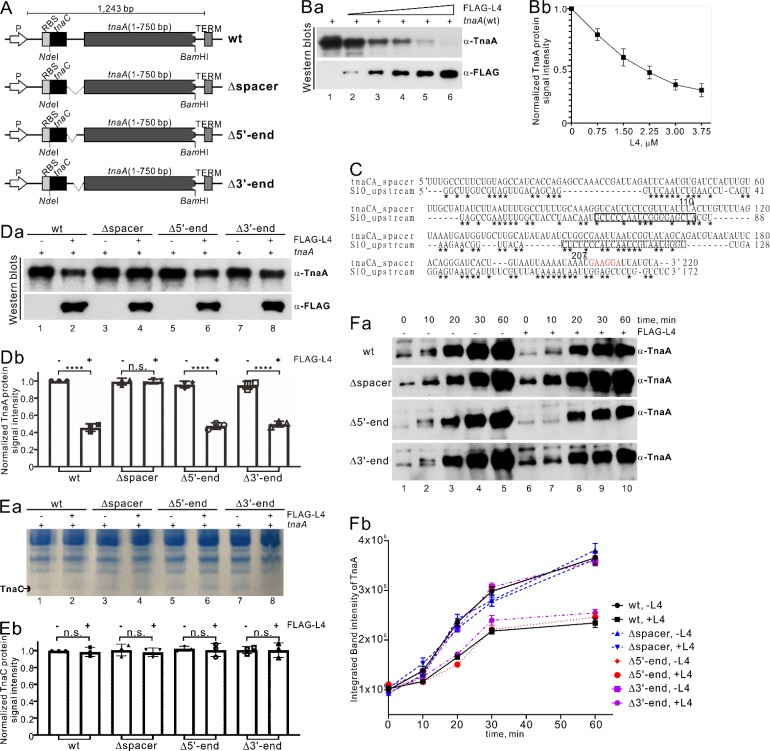
L4 downregulates TnaA but not TnaC protein synthesis in an *in vitro* transcription/translation assay. (A) Schematic representation of a synthetic *tna* operon for *in vitro* transcription and translation with the PURExpress *in vitro* protein synthesis kit (New England BioLabs, UK). The upstream T7 promoter (P), ribosome binding site (RBS), NdeI and BamHI cloning sites, and the T7 terminator (TERM) downstream of the stop codon are indicated, respectively. Internal deletion variants (indicated by Δspacer, Δ5′-end, and Δ3′-end) of the *tnaC-tnaA* spacer in the synthetic *tna* operon were generated as described in the Materials and Methods and further used for *in vitro* transcription/translation in the absence and presence of FLAG-L4. (B) L4 downregulates TnaA protein synthesis. Shown are Western blot analysis (a) and quantitative data (b) for TnaA protein produced from the wild-type (wt) synthetic *tna* operon and FLAG-L4 upon detection by TnaA polyclonal antibody (α-TnaA) or FLAG peptide monoclonal antibody (α-FLAG) on 10% SDS-PAGE. (a) Increasing concentrations of FLAG-L4 (0.75, 1.5, 2.25, 3, and 3.75 μM, lanes 2 to 6) inhibited TnaA translation. Lane 1 shows the reaction control (without FLAG-L4). (C) Comparison of RNA sequences of *tnaC-tnaA* spacer (220 nt) and S10 upstream regions (172 nt) by means of a CLUSTAL O (1.2.4) multiple sequence alignment (27). Solid and dotted lines box nucleotides involved in RNA binding of L4 with S10 UTR to regulate transcription and both transcription and translation, respectively (11, 53, 57). Asterisks indicate identical nucleotides. The Shine-Dalgarno (SD) sequence of TnaA is shown in red. (D to F) The inhibitory effect of L4 on the synthesis of TnaA but not TnaC by the synthetic *tna* operon. Shown are Western blots of TnaA (Da) and a 15% Bis-Tris polyacrylamide gel (Ea) of TnaC produced from the wt and various deletion constructs (see panel A) of the synthetic *tna* operon in the absence of L4 (lanes 1, 3, 5, and 7) compared with a positive-control plasmid in the presence of L4 (lanes 2, 4, 6, and 8). The reaction was stopped using 2× SDS loading dye 60 min after adding template DNA, and we then conducted 10% SDS-PAGE and detection with antibodies against FLAG peptide and TnaA or a 15% Bis-Tris polyacrylamide gel to resolve the very small molecular weight TnaC protein (∼3 kDa). The concentration of FLAG-L4 was 1.5 μM. The TnaC protein was further confirmed by peptide sequencing. (Db and Eb) Quantification of the data obtained in panels Da and Ea is presented, and TnaA and TnaC protein levels were compared from different spacer deletion constructs as indicated. *n* = 3; mean ± SEM; ****, *P* < 0.0001; n.s. represents no significance after one-way ANOVA and Tukey’s *post hoc* test. (F) Time-dependent inhibition of TnaA protein synthesis by L4. Shown are Western blot analysis (a) and quantitative data (b) of TnaA protein produced from the WT and various deletion constructs (see panel A) of the synthetic *tna* operon in the absence of L4 (lanes 1 to 5) compared with a positive-control plasmid in the presence of L4 (lanes 6 to 10). The concentration of FLAG-L4 was 1.5 μM. Aliquots for SDS-PAGE were withdrawn every 10 min for 60 min after addition of template DNA, and then we conducted 10% SDS-PAGE and detection with antibodies against TnaA. The faint bands in lane 1 are from nonspecific binding by use of polyclonal anti-TnaA. Error bars indicate ±SEM.

To investigate the possible role of L4 in downregulating translation of the *tna* operon through its binding to the *tnaC-tnaA* transcribed spacer region, we compared TnaA immunoblot signals after protein synthesis from two plasmids (a positive and negative control) to determine signal specificity for TnaA in a cell-free transcription/translation system. We first confirmed that TnaA protein synthesis occurs from the plasmid carrying the synthetic *tna* operon (positive control) and not from a negative-control plasmid encoding E. coli dihydrofolate reductase (DHFR) (Fig. S3Aa, lower panel) in which no TnaA was generated (Fig. S3Ab, lane 2 versus 1). We found that TnaA protein synthesis decreased in the presence of increased purified FLAG-L4 abundance (Fig. 5Ba, lanes 2 to 6; quantification data in Fig. 5Bb).

Because r-protein L4 regulates translation by binding to an mRNA sequence/structure in the S10 operon that mimics the binding sites for the r-protein on rRNA, we investigated whether some kind of similar molecular mimicry also existed in the *tnaCA* spacer RNA. We compared the RNA sequences of the *tnaC-tnaA* spacer (220 nt) and the S10 upstream region (172 nt) by means of CLUSTAL O (1.2.4) multiple sequence alignment (27), and found that the *tnaC-tnaA* spacer and S10 upstream regions exhibit >50% identity (Fig. 5C), suggesting that molecular mimicry may be the mechanistic basis for L4 binding to the *tnaC-tnaA* spacer region.

We constructed different deletion mutants of the *tnaC-tnaA* spacer region (but with retention of the ribosomal binding sequences upstream of *tnaA*) and performed *in vitro* translation studies. The resulting Δspacer (as a negative control), Δ5′-end (deletion of the 5′ half of the spacer region, from nt 1 to 110), and Δ3′-end (deletion of the 3′ half of the spacer region, from nt 111 to 207) mutants (Fig. 5A) allowed us to determine the RNA-interacting region of L4 protein that regulates TnaA translation. Our results show that the wild type and the Δ5′-end and Δ3′-end mutants exhibited similarly reduced TnaA translation in the presence of L4 (Fig. 5Da, compare lanes 2, 6, and 8 with lanes 1, 5, and 7; quantification data in Fig. 5Db). As expected, TnaA translation was not reduced in the presence of L4 for the Δspacer mutant (Fig. 5Da, compare lanes 3 and 4 with lanes 1 and 2; quantification data in Fig. 5Db). We confirmed the reduction of TnaA synthesis by means of time-dependent experiments (Fig. 5Fa, compare lanes 1 to 5 with lanes 6 to 10; quantification data in Fig. 5Fb).

We hypothesized that if L4 binds within the *tnaC-tnaA* transcribed spacer region to inhibit TnaA translation, L4 should not affect translation of the leader peptide TnaC that lies upstream and proximal to the transcribed spacer. Indeed, *in vitro* translation of TnaC occurred for all constructs, both in the presence and absence of FLAG-L4 (detected in a Bis-Tris acrylamide gel [Fig. 5E]). The Coomassie blue-stained gel revealed migration of a protein of molecular weight similar to TnaC (2.894 kDa) under both conditions, which we subsequently confirmed as TnaC by tandem mass spectrometry (MS-MS) protein sequencing. Additionally, we assessed translation of DHFR from a control plasmid containing the DHFR template in the presence of the highest concentration of L4 (3.75 μM) and found that L4 does not have any effect on DHFR translation (Fig. S3B). Thus, translational inhibition of TnaA by L4 in the *tna* operon is very specific and does not affect translation of the TnaC leader peptide.

### Binding of L4 in the *tnaC-tnaA* transcribed spacer results in structural alteration of the RNA.

Our *in vitro* RNA binding and *in vitro* translation assays demonstrated that L4 binds to the *tnaC-tnaA* transcribed spacer region in the *tnaCAB* operon to regulate TnaA translation. Regulatory regions of bacterial RNA undergo conformational changes in response to RNA-protein interactions (28).

**FIG 6:**
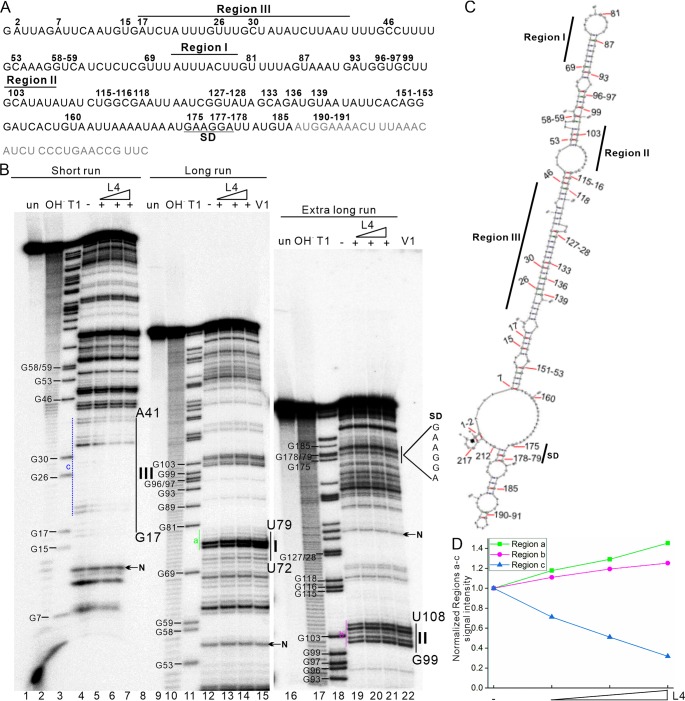
Structural probing of the 5′-end-labeled *tnaC-tnaA* transcribed spacer in the absence and presence of FLAG-L4. (A) DNA template (239 bp) containing the *tnaC-tnaA* spacer was transcribed under the T7 promoter to generate a 220 nt final RNA product. The sequence of the final 220-nt RNA product (with addition of a G at the 5′ end by RNA polymerase during *in vitro* transcription) is shown. The Shine-Dalgarno (SD) sequence of TnaA is underlined. Affected regions I, II, and III in the footprint determination are indicated in the primary sequence. The numbers at the top of the primary sequence represent the RNase T1 cleavage sites between guanosine 3′-phosphate residues and the 5′-OH residues of adjacent nucleotides, as shown in panel B (lanes 3, 11 and 18). The gray font in the primary sequence from the 3′ end shows the beginning of the coding sequence for TnaA. (B) The RNA was further 5′ end labeled with [γ-^32^P]ATP and purified from the denatured acrylamide gel. The 5′-end-labeled RNA (30,000 cpm, ∼2 ng) was then incubated at room temperature in the absence (−) or presence (+) of L4 (5.5 μM) for binding, before conducting RNase V1 digestion (double strand-specific cleavage; lanes 5 to 7, 13 to 15, and 20 to 22) and separation on an 8% polyacrylamide–8 M urea gel. The 5′-end-labeled RNA fragments generated by alkaline and RNase T1 hydrolysis (OH and T1, respectively) are shown. The experiments were carried out on untreated RNA (un) or in the presence of increasing concentrations of purified FLAG-L4. Regions I, II, and III affected by L4 in the footprint determination are indicated (also in panel C). Regions a, b, and c used for quantification are indicated with colored lines. Bands (N) used for normalization of the phosphor image signals of regions a, b, and c are indicated. (C) The 220 nt RNA secondary structure predicted by RNAfold (56) (dG = −44.05). (D) Quantification of regions a, b, and c affected by L4 in the footprint determination.

We therefore investigated whether there were conformational changes to the *tnaC-tnaA* spacer RNA induced by RNA-protein interactions in the presence of L4 (Fig. 6A). Secondary structure predictions of the *tnaC-tnaA* spacer reveal highly paired regions and a helical structure (Fig. 6B and C). We used RNase V1 (which preferentially cleaves paired regions of structured double-stranded RNA) to probe a fragment of the *tnaC-tnaA* spacer (220 nt) spanning the 35th nucleotide of the 5′ end of the spacer RNA and the 33rd nucleotide of the *tnaA* coding region, thereby retaining the ribosomal binding sequences upstream of *tnaA*. Transcribed RNA was labeled at the 5′ end and then probed with RNase V1 in the absence (Fig. 6B, lanes 4, 12, and 19) or presence (Fig. 6B, lanes 5 to 7, 13 to 15, and 20 to 22) of increasing concentrations of FLAG-L4. Denaturing polyacrylamide gel electrophoresis revealed that the region between nucleotides G17 to A41 of the *tnaC-tnaA* spacer fragment is not accessible for RNase V1 cleavage because of L4 binding (Fig. 6B, marked as region III; quantification data in Fig. 6D). Regions corresponding to nucleotides U72 to U79 and G99 to U108 (stem-loop regions) were highly prone to RNase V1 cleavage in the presence of L4 (Fig. 6B, marked as regions I and II, respectively; quantification data in Fig. 6D), perhaps because conformational alteration of region III rendered regions I and II more accessible to RNase VI cleavage (Fig. 6C). Together, these data show that structural changes induced by L4 binding in the *tnaC-tnaA* transcribed spacer region may result in decreased TnaA synthesis/translation levels.

### E. coli fine-tunes posttranscriptional expression of its TnaA protein during early stationary growth phase.

**FIG 7:**
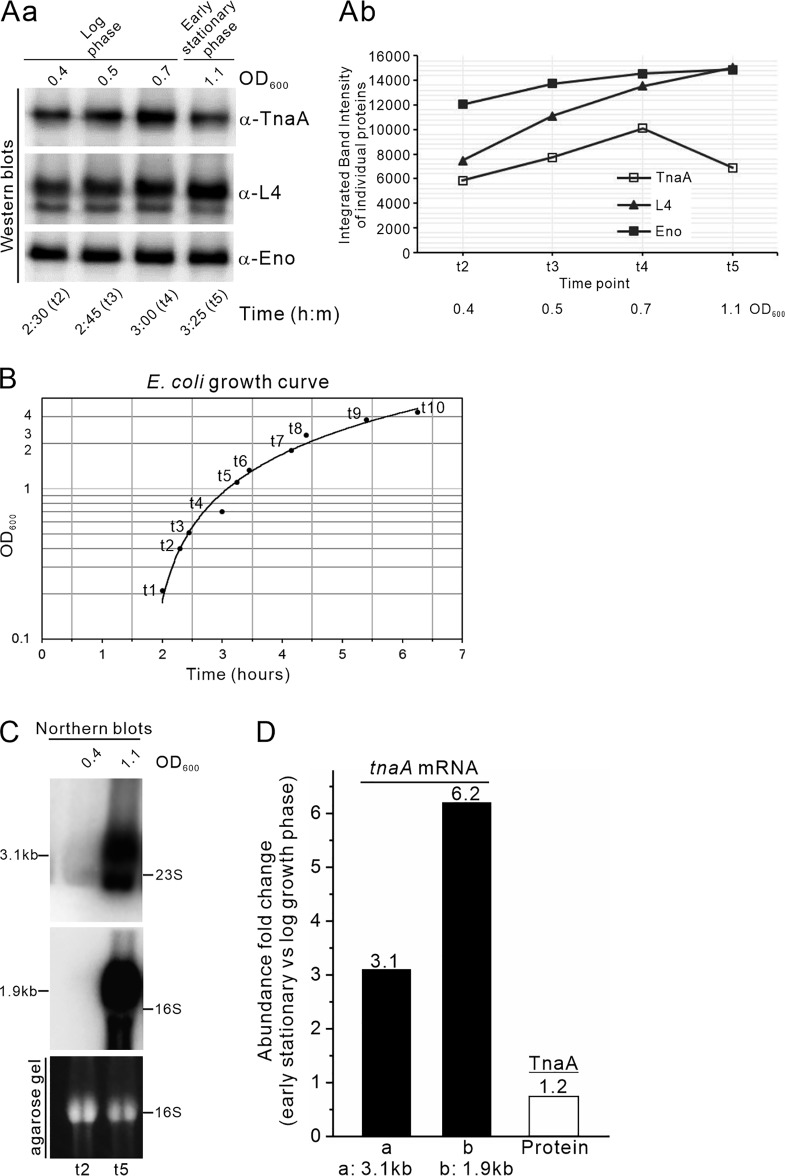
Posttranscriptional regulation of the *tna* operon during early stationary-phase growth can fine-tune TnaA levels. (Aa) TnaA protein expression levels. N3433 bacterial cells were grown in LB medium at 32°C (with 180 rpm) and 1 ml of culture was harvested for Western blot analysis (10% SDS-PAGE) at the indicated OD_600_ (represented at the corresponding time points in the parentheses starting from t2 to t5) using anti-TnaA, -L4, and -Eno antibodies. (Ab) Quantitative data for TnaA, L4 and enolase (Eno) expression levels shown in the Western blot of panel Aa. Western blots of endogenous TnaA, L4, and Eno were detected from cultured cells at the indicated t2 (OD_600_ = ∼0.40) to t5 (OD_600_ = ∼1.1) growth phase. The integrated band density of individual proteins obtained from the Western blot signals for antibody detection against TnaA, L4, or Eno was plotted for each time point (t2 to t5) as shown. (B) Semilogarithmic plot of E. coli strain N3433 as observed for more than 6 h after culture inoculation (t0, OD_600_ value of ∼0.02). The time point t1 indicates a culture sample collected after 2 h of inoculation (OD_600_ of ∼0.2), and culture samples were collected thereafter at different time points (t2 to t10). (C) Aliquots (50 ml) of bacterial cells from log phase (t2, OD_600_ = ∼0.4) to early stationary phase (t5, OD_600_ = ∼1.1) were collected and their *tna* transcripts were examined by Northern blotting analysis. The 3.1-kb and 1.9-kb *tna* RNA transcripts, as well as 16S rRNA, are shown. The OD value of each sample is shown above the gel. (D) Fold change in abundances of *tnaA* mRNA and TnaA protein. To calculate fold change, the band intensity of an individual *tnaA* transcript detected in panel C, i.e., 3.1 kb or 1.9 kb, and the TnaA protein detected in panel A were quantitated using VisionWorks LS software.

We assessed *in vivo* expression of TnaA protein during two stages of E. coli N3433 growth (log and early stationary phase) (Fig. 7A and B). We showed that *tnaA* mRNA and TnaA protein levels do not vary in a coordinated way. During log phase growth, *tnaA* mRNA expression was much lower than for early stationary phase (Fig. 7C, compare *tnaA* mRNA levels for OD_600_ of ∼0.4 at t2 and OD_600_ of ∼1.1 at t5). Quantitative results showed that levels of the *tna* 1.9- and 3.1-kb mRNA species in early stationary growth phase were increased by 3.1- and 6.2-fold (Fig. 7D), respectively, compared to log phase. However, corresponding TnaA protein levels did not increase concomitantly by 9-fold (only by 1.2-fold [Fig. 7D and Ab]) during early stationary growth phase compared to log phase. These data provide evidence that posttranscriptional regulation can fine-tune TnaA protein expression in response to cell growth.

## DISCUSSION

Certain r-proteins have been reported as having extraribosomal functions relating to various biological processes or functions in mammals (29, 30), yeast (31–33), and bacteria (3, 4, 34). However, there are some functions of r-proteins unrelated to ribosome function that remain to be determined (2, 3, 35). r-proteins are an integral part of the translation machinery and are responsible for stabilizing rRNA structure (36, 37). However, some r-proteins in their free form exert extraribosomal functions (such as in translational repression, transcriptional regulation, mRNA stability, DNA repair and replication, and phage RNA replication) by binding RNA and interacting with specific cellular proteins (3). In this study, we have identified a novel extraribosomal function for the r-protein L4. We have shown that ectopically expressed L4 binds to the *tnaC-tnaA* transcribed spacer region, induces conformational changes in the spacer RNA, and affects adjacent *tnaA* translation, resulting in decreased levels of TnaA protein. Our data suggest that L4 tends to bind to *tnaC-tnaA* mRNA by means of molecular mimicry (Fig. 5). We have also shown that endogenous L4 binds to the *tnaC-tnaA* spacer RNA transcripts in early stationary phase but not in exponentially growing E. coli cells and that *tnaA* mRNA abundance and TnaA protein levels are not positively correlated. The increase in early stationary-phase *tnaA* mRNA levels were 6-fold higher in early stationary phase than in log phase, but TnaA protein levels did not change in parallel. Our study reveals a possible mechanism by which binding of L4 to the *tnaC-tnaA* spacer mRNA may interfere with translation, thereby inhibiting TnaA protein synthesis throughout cell growth. Our study also suggests that L4, which is known to regulate its own operon, is a multifaceted protein that can precisely control the levels of various proteins through its binding affinity for RNA or protein, allowing E. coli to survive diverse physiological conditions.

L4 regulates RNase E activity and stabilizes transcripts to increase their protein levels (4). It also binds to cellular RNAs such as S10-UTR to repress translation by interfering with translation initiation (7, 11). It was not known previously that the *tnaA* transcript is regulated by L4. The half-life of the 3.1-kb or 1.9-kb *tnaA* transcript increased by only 25% to 50% upon ectopic L4 expression (Fig. 2C). Since the N3433 strain we used in this study contained *relA1* and *spoT1* mutations that would tend to reduce ppGpp levels, it is possible that greater regulation would occur in a wild-type strain able to produce high levels of ppGpp. However, despite the increased abundance of *tna* transcripts in our system, TnaA protein levels were reduced in the presence of ectopically expressed L4. We show that free L4 downregulates translation but not transcription of TnaA (Fig. 3), with the former function dependent on interactions of L4 with *tnaC-tnaA* spacer RNA. We detected an increase in the stability of *tna* transcripts (the 3.1-kb and 1.9-kb RNA species) when temperature-sensitive *rne* [*rne*(Ts)] N3431 cells were shifted to a nonpermissive temperature (44°C) compared to cells maintained at a permissive temperature (32°C) (Fig. 2D and E). Moreover, TnaA protein levels increased (Fig. 1C) with an increase in *tna* transcript abundance under those conditions (Fig. 2D and E). These results indicate that E. coli uses the extraribosomal function of L4 to respond to certain growth conditions. In this study, we have demonstrated that endogenous L4 binds to the *tnaC-tnaA* spacer during early stationary growth of E. coli (Fig. 4).

We generated constructs with various deletions in the *tnaC-tnaA* spacer region and used them to establish the L4-RNA interaction region by *in vitro* transcription/translation (Fig. 5). We found that deletion of either the 5′ or 3′ half of the spacer region between *tnaC* and *tnaA* resulted in a similar reduction in TnaA translation in the presence of L4 compared to WT, suggesting that a L4 binding site(s) is located within both halves of the spacer region. The process by which L4 binding to the *tnaC-tnaA* spacer impacts downstream *tnaA* mRNA translation, ribosome occupancy, and ribosome density is still unknown.

Our results highlight the importance of the extraribosomal function of L4 in translational regulation of TnaA, which may be involved in the physiological transition of E. coli cells into early stationary phase. TnaA primarily degrades l-tryptophan to indole, pyruvate, and ammonia (38). It has also been shown to exhibit l-cysteine desulfhydrase (CD) activity to catalyze degradation of l-cysteine to pyruvate, ammonia, and sulfide (39, 40). During the exponential/early stationary phase of growth, E. coli may preferentially maintain low levels of TnaA protein, despite there being high levels of corresponding mRNA, since amino acids like tryptophan and cysteine are needed to sustain cell growth during the stationary or late stationary phases of E. coli cell growth. During the early stationary phase of bacterial cell growth, free-form L4 may bind to the *tnaC-tnaA* transcribed spacer region to negatively control subsequent TnaA protein translation, thereby maintaining low levels of TnaA protein and preventing rapid degradation of tryptophan/cysteine. This layer of regulation may be necessary since tryptophan levels in the cell are known to control transcription of the *trp* operon for tryptophan synthesis via a negative feedback loop (41, 42). Furthermore, ribosomes undergo degradation during the stationary phase of cell growth (43, 44), which can generate free-form L4 to fine-tune TnaA protein levels.

**FIG 8:**
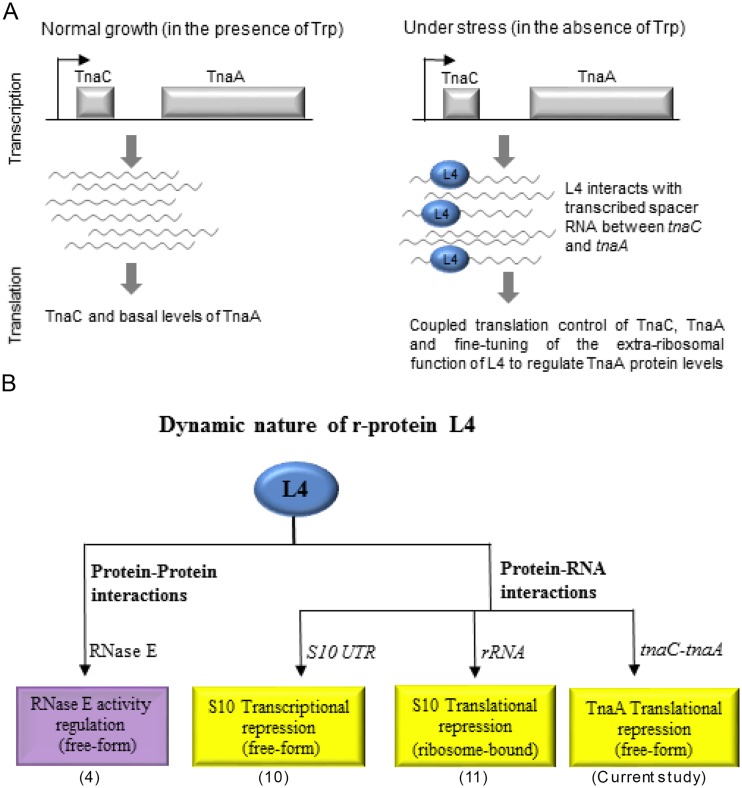
Schematic representation of the extraribosomal function of the r-protein L4 in TnaA translation and an overview of the multifaceted nature of L4 protein. (A) Posttranscriptional regulation of TnaA translation under stress conditions compared to normal growth. The *tnaC-tnaA* transcribed spacer RNA is the binding site of the r-protein L4 responsible for regulating translational levels of TnaA. (B) The multifaceted nature of the r-protein L4 as represented by L4 protein-protein or L4 protein-RNA interactions. References for the various functions of L4 are indicated below.

RNA-binding proteins are important regulators of gene expression at the posttranscriptional level. The RNA-binding activity of r-proteins and their ability to exist in a free-form state render them suitable for performing extraribosomal functions. Here, we present a previously undocumented extraribosomal function for the r-protein L4 that is important for responses to physiological growth conditions (Fig. 8A). Our study further confirms the multifaceted nature of L4, as summarized in Fig. 8B, wherein L4 can interact with rRNA (ribosome-bound form) (11), bind to its own S10-UTR to control transcription and translation (free-form) (10), and bind to the *tnaC-tnaA* transcribed spacer region to inhibit TnaA translation (this study). L4 also interacts with specific cellular proteins, including RNase E (14). Thus, L4 can distinguish its specific transcripts in a cellular pool of mRNAs through one or more of its diverse functional domains and subsequently execute its unique extraribosomal roles either alone or in a complex cellular machinery.

Tryptophan-induced transcription antitermination is known to regulate the *tna* operon in E. coli (45, 46). This mechanism requires synthesis of the tryptophan-containing leader peptide TnaC, TnaC-tRNA(Pro), and an inducing (excess) level of tryptophan. The nascent TnaC-peptidyl-tRNA(Pro) is retained in the ribosome, resists peptidyltransferase cleavage, stalls at the TnaC stop codon, and prevents Rho factor from entering the *rut* binding site on the *tnaC* transcript, thereby preventing transcription termination (47). The downstream-paused RNA polymerase restores transcription of the *tna* operon genes (*tnaA* and *tnaB*). The *tnaC-tnaA* spacer region, which separates the *tnaC* stop codon from the *tnaA* start codon, contains several transcriptional pause sites that can serve as regulated sites of Rho-dependent transcription termination (46). In the present study, we show that the *tnaC-tnaA* spacer sequence serves as a site for posttranscriptional modulation of TnaA translation. Abundance of the 3.1-kb and 1.9-kb *tna* transcripts increased in N3433 cells during the early stationary phase of growth, but there was almost no change in concomitant levels of TnaA protein (Fig. 7).

Our discovery, together with the previously known regulatory mechanisms of the *tna* operon, provide multiple strategies for bacterial cells to prevent accumulation of high levels of tryptophanase. High levels of tryptophanase would degrade cell-synthesized tryptophan, resulting in a tryptophan deficiency that would induce the tryptophan operon, which might inhibit cell survival under various physiological conditions.

## MATERIALS AND METHODS

### Bacterial strains, plasmids, and antibodies.

E. coli K-12 strain N3433 (45) encoding full-length RNase E was used to determine RNA stability, RNase E-dependent regulation of protein expression by L4, and detection of endogenous RNase E and Flag-L4. E. coli strain BL21(DE3) (*rne131*) (46) encoding a truncated version of RNase E (amino acids 1 to 585) was used to purify Flag-L4. RNase E temperature-sensitive [*rne*(Ts)] N3431 strain (47, 48) at the permissive (32°C) and nonpermissive (44°C) temperatures was used for the analysis of transcripts by Northern blotting.

The plasmid pPWflagL4 was constructed as described previously (4) and was used to express Flag-L4. E. coli ASKA JW3686 clone (49) encoding *tnaA* tagged with histidine at its N terminus and having the IPTG-inducible promoter pT5/lac was used for TnaA protein purification to generate polyclonal rabbit antibody. RNase E polyclonal antibody was generated previously in our laboratory (50). Monoclonal antibodies for FLAG-M2 (Sigma, USA) and RpoS (Abcam, USA) were purchased commercially. Antibodies for Lon and CspE were a generous gift from Susan Gottesman (NIH, USA) and Narendra Jawali (BARC, India), respectively.

### Northern blot analysis.

Bacterial cell cultures were grown at 32°C to an OD_600_ of ∼0.3 and total RNA was isolated from the steady-state cell cultures after ectopic expression of L4 (induced with 0.5 mM IPTG) for 30 min. To analyze the effect of L4 expression on *tnaA* mRNA stability, cell cultures were collected at different time points after the addition of rifampin (0.5 mg/ml) as described previously (4). The temperature was increased from 37°C to 44°C for 30 min to inactivate RNase E in the *rne*(Ts) mutant before rifampin treatment. Total RNAs were fractionated on 0.8% agarose-formaldehyde gels. The fractionated RNA was further transferred to Zeta-Probe blotting membranes (Bio-Rad) and separately hybridized with RNA probes designed from different regions of the *tna* operon (Fig. 2A). Probe labeling with ^32^P, probe synthesis using an *in vitro* transcription kit (MAXIscript, Ambion), Northern signal visualization, and quantification were conducted as described previously (4) and according to the manufacturer’s instructions. RNA half-lives were determined by nonlinear regression curve fitting (one phase decay) using GraphPad Prism. We used following the parameters: (i) least-squares (ordinary fit); (ii) confidence level of 95%; (iii) asymmetrical (likelihood) CI; (iv) goodness of fit quantified with R square; and (v) convergence criteria, medium. DNA templates for transcription of individual RNA probes were generated by PCR using gene-specific primers (see Table S2 in the supplemental material).

### SDS-PAGE and Western blot analysis.

Total lysates were prepared from the control plasmid (pPWflag only) and from the N3433 strain after L4 expression induced with IPTG (0.5 mM) for 30 min. Electrophoresis of protein samples was carried out as described previously (51). For Western blot analysis, proteins were separated in 10% SDS polyacrylamide gels and transferred onto PVDF membranes using a semidry apparatus (ATTO Corp.). After blocking, the membranes were cut into smaller pieces and then probed with specific antibodies for TnaA, RNase E, Flag-L4, and TolB (as a loading control). Incubation of secondary anti-IgG antibodies, as well as detection and visualization of protein bands, was conducted as described previously (4). Assays were performed with primary rabbit (anti-L4, anti-RNase E, anti-TnaA, anti-CspE, anti-Lon, and anti-TolB), and mouse (anti-M2 for FLAG-L4 and anti-RpoS) antibodies.

### Protein purification of Flag-L4 and His-TnaA.

Protein purification of Flag-L4 (∼23 kDa) was performed as described previously (4). Briefly, clear lysate from E. coli strain BL21(DE3) (*rne131*) containing Flag-tagged protein was loaded on an anti-M2 affinity column (Sigma) preequilibrated in buffer A (50 mM Tris-HCl [pH 8.0], 0.15 M NaCl). Protein was purified using 1 ml of a solution of synthetic FLAG oligopeptide (1 mg/ml) (PAN Laboratory, Stanford University). To generate tryptophanase (TnaA, 52.7 kDa) polyclonal antibody, we used the ASKA JW3686 clone (49) for TnaA protein purification. Bacterial culture, induction with IPTG (0.1 mM), and His-tagged protein purification was performed as per the ASKA clone description (49). Briefly, clear cell lysates obtained after cell lysis were loaded onto a Ni-NTA affinity chromatography column (Thermo Fisher Scientific, USA) and washed with washing buffer (20 mM Tris-HCl [pH 8.0], 0.5 M NaCl, 5 mM imidazole) until the OD_280_ of flow-through reached ≥0.5. Finally, proteins were eluted with buffer (20 mM Tris-HCl [pH 8.0], 0.5 M NaCl, 250 mM imidazole). The highly purified protein was dialyzed and concentrated using an Amicon ultra column (Millipore). Purified TnaA protein quality (52.7 kDa) was checked on 10% SDS-PAGE. Purified TnaA protein was then used for rabbit immunization to generate TnaA polyclonal antibody under the supervision of the IMB Animal Facility (Academia Sinica) using a standard procedure.

### Mobility gel shift assay.

### 

To examine RNA-protein interactions between the *tnaC-tnaA* transcribed spacer region and the r-protein L4, we prepared DNA template of this transcribed spacer region using specific PCR primers (Table S2), with a T7 promoter incorporated into the reverse primer. RNA template was *in vitro*-transcribed (261 nt comprised of 20 nt upstream and 20 nt downstream of the spacer [220 nt] plus one “G” added by RNA polymerase from the 5′ end) and internally labeled with [α-^32^P]UTP in an *in vitro* transcription assay as per the vendor’s instructions (MAXIscript T7 transcription kit; Ambion). RNA templates for S10-UTR, 23S rRNA domain I and L4 nontarget *rpsO*-UTR were also prepared using gene-specific primers (Table S2) before undergoing *in vitro* transcription and internal labeling to generate final products of 172, 182, and 164 nt, respectively. Mobility gel shift assays were performed as described previously (52). Briefly, full-length radioisotope-labeled *tnaC-tnaA* transcript was purified from the denaturing gel and incubated in the absence of or increasing concentrations of FLAG-L4. Reaction mixtures were incubated at 30 °C for 20 min and were stopped by adding nondenaturing sample loading buffer (1 mM EDTA, 50% [vol/vol] glycerol, 0.025% xylene cyanol, and 0.025% bromophenol blue). Reaction products were analyzed by electrophoresis on nondenaturing 5% polyacrylamide gels followed by exposure to X-ray film (Kodak, Thermo Fisher Scientific, USA).

Binding affinity analysis of the L4-*tnaC-tnaA* complex was performed with a fixed concentration of ^32^P *tnaC-tnaA* spacer RNA (30,000 cpm, ∼2 ng) and increasing concentrations of L4 protein (0.7, 1.4, 2.1, 2.7, 4.1, and 5.5 μM). The RNA bands in the gel were detected using a Typhoon FLA 9000 Biomolecular Imager (GE Healthcare). The integrated band intensity for L4-*tnaC-tnaA* complex was quantitated by VisionWorks LS software based on the intensity of signals obtained by scanning the gel. For the *K_d_* calculation, we used nonlinear regression performed in GraphPad Prism software to fit the data to a simple binding equation.

### β-Galactosidase activity assay.

To create the transcriptional fusion construct of the *tna* promoter (Ptna-*galK*’-’*lacZ*’), we used pMU575 vector (single copy number *Inc*W replicon with a promoterless *lacZ* gene and trimethoprim resistance) (22). pMU575 is a *galK’lacZ’YA*’ fusion vector in which the amino terminus of the *galK* gene, containing all the translational start signals but no promoters, is fused in phase with the eighth codon of the *lacZ* gene. The *tna* promoter sequence (123 nt upstream from the transcriptional start site in the *tna* operon) was amplified using specific forward (including a BamHI site) and reverse (including a HindIII site) primers, generating a PCR product of ∼150 bp (Table S2). PCR product and the vector pMU575 were then digested with BamHI and HindIII restriction enzymes. Reaction mixtures after restriction digestion were cleaned, ligated, and transformed into DH5α. To create the translational fusion constructs of the *tna* promoter without (Ptna-*tnaC’-’lacZ*’) or with the *tnaC-tnaA* spacer (Ptna-*tnaC-tnaA’-’lacZ*’), we used pMU2386 derived from pMU525 that lacks the appropriate translation start signal (23). The *tna* promoter sequence of the *tna* operon (nt −123 to +36 for Ptna-*tnaC’-’lacZ*’ or −123 to +288 for Ptna-*tnaC-tnaA’-’lacZ*’) was amplified using specific forward (including a HindIII site) and reverse (including a BamHI site) primers (Table S2), generating PCR products of ∼180 and 441 bp that were then digested with BamHI and HindIII restriction enzymes and cloned into pMU2386. Positive transformants for the *tna* transcriptional and translational fusion constructs were screened against trimethoprim (40 μg/ml) and positive sequences were confirmed by sequencing using the LacZ-Rseq primer (Table S2) designed from the β-galactosidase gene. The *tna* transcriptional and translational fusion constructs were then separately cotransformed with either pBAD-Flag only (control plasmid) or pBAD-Flag-L4 plasmids in strain N3433, and transformants were selected against ampicillin (50 μg/ml) and trimethoprim (40 μg/ml). The respective positively transformed bacterial cells were first grown overnight and then subcultured in LB medium to grow to OD_600_ of 0.3 to 0.4 before being induced with 0.2% arabinose for L4 expression for 30 min at 32°C × 180 rpm. For the control experiment, we used N3433 cells without vectors, which contain a *lacZ43*(*Fs*) mutation and cannot produce β-d-galactosidase. We harvested 1.0 ml of cultures and subjected them to a β-galactosidase assay, as described previously (53). β-Galactosidase activity (in Miller units) was calculated every 10 min for 80 min after adding the β-galactosidase substrate *o*-nitrophenyl-β-d-galactopyraniside (ONPG) and then plotted.

### Polyclonal L4 antibody coimmunoprecipitation assay.

E. coli N3433 cell cultures (100 ml) were grown at 30°C × 180 rpm in LB medium and cultures were collected at different cell growth stages (OD_600_ = 0.4, 1.4, and 1.8). Cell pellet lysis and coimmunoprecipitation with polyclonal L4 antibody were performed as described previously (54) using protein A-Sepharose beads (Amersham Pharmacia) except that protein elutions were performed with 300 μl of 0.1 M glycine (pH 2.4). The eluted proteins were immediately neutralized by adding 3 M Tris pH 8.8 (9 μl) and 5 M NaCl (6 μl). The RNA was isolated from the copurified protein complex of L4 by sequential extraction with phenol, phenol-chloroform, and chloroform. The aqueous phase was mixed with 0.1 volume of 3 M sodium acetate and then isopropanol-precipitated. The pellet was suspended in 20 μ1 of TE buffer (10:1 mM), treated with DNase I to remove DNA contamination, repurified with phenol-chloroform, and then subjected to chloroform treatment. The purified RNA (100 ng) was reverse-transcribed using a First-strand cDNA Synthesis kit (Invitrogen) as per the manufacturer’s instructions. Then, DNA template-specific primers for *tnaC-tnaA* spacer, S10-UTR, and *rpsO*-UTR (Table S2) were directly used for gene-specific PCR amplification using cDNA as a template. The amplified products were resolved on an agarose gel and stained with ethidium bromide dye.

### Cell-free transcriptional/translation activity assay.

A synthetic *tna* operon was constructed from the DHFR control plasmid provided with the PURExpress *in vitro* protein synthesis kit (New England BioLabs, UK). This synthetic *tna* operon (comprising 1,243 bp harboring *tnaC*, the transcribed spacer region, and the initial 750 bp of *tnaA*) was constructed using specific primers (Table S2) after excising the DHFR DNA with appropriate restriction enzymes (NdeI and BamHI). Internal deletion variants (Δspacer, Δ5′-end, and Δ3′-end) of the *tnaC-tnaA* spacer were generated by inverse PCR (55) using specific primers (Table S2). L4-mediated inhibition of TnaA translation was achieved using plasmid DNA (containing the synthetic *tna* operon) as the template and according to the manufacturer’s instructions (PURExpress *in vitro* protein synthesis kit; New England BioLabs, UK). Fixed (1.5 μM) or increasing concentrations (from 0.75 to 3.75 μM) of FLAG-L4 were added to the *in vitro* translation assay. Reactions were stopped by adding SDS-PAGE sample loading dye and further fractionated on a 10% SDS denaturing polyacrylamide gel. The levels of TnaA and L4 in the *in vitro* translation assays were detected by Western blotting using the specific antibodies described above in “SDS-PAGE and Western blot analysis.”

### Structural probing of *tnaC-tnaA* transcribed spacer RNA.

DNA template (239 bp), corresponding to 186 bp of the transcribed spacer region between *tnaC* and *tnaA* and 33 bp of the coding region of *tnaA*, was amplified using specific primers (Table S2). The DNA template was transcribed under the T7 promoter (20 bp) to generate RNA transcripts of 220 nt in length (with an additional “G” added by RNA polymerase) (Ambion). The transcript was further purified to remove unincorporated nucleotides using G50 spin columns (GE Healthcare). We dephosphorylated the 5′ end of the RNA using alkaline phosphatase and 5′ end labeled it with [γ-^32^P]ATP before purifying it from a denaturing acrylamide gel. The 5′-end-labeled *tnaC-tnaA* RNA was incubated in the absence or presence of L4 at room temperature for r-protein binding, before being subjected to enzymatic digestion. RNase T1 (5 U/μl; Ambion), which cleaves single-stranded regions after G, was titrated to obtain a 1:800 dilution and incubated at 50 °C for 15 min in 1× sequencing buffer (20 mM sodium citrate [pH 5], 1.0 mM EDTA, 7 M urea). RNase V1 (0.1 U/μl; Ambion), which cleaves paired regions, was titrated to obtain a 1:100 dilution and incubated at room temperature (25°C) in 1× RNA structural buffer (100 mM Tris [pH 7], 1.0 M KCl, 100 mM MgCl_2_; Ambion). The 5′-end-labeled RNA was treated with alkaline hydrolysis buffer (50 mM sodium carbonate [pH 9.2], 1.0 mM EDTA; Ambion) at 95°C for 3 min to generate an alkaline ladder. Reactions were stopped with an equal volume of loading buffer (95% formamide, 20 mM EDTA, 0.05% bromophenol blue, 0.05% xylene cyanol) and further fractionated on 8% polyacrylamide–8 M urea gels, run at 26 W for various time frames, and visualized by PhosphorImager (FLA-5000; FUJIFILM). The secondary structure of RNA used for structural probing was predicted using the Mfold web server (56).

### Quantification and statistical analysis.

Integrated band intensity was quantitated using VisionWorks LS software based on the intensity of signals obtained by scanning the gels, membranes, or phosphor image plates. Optical density (OD) for β-galactosidase activity assays was obtained using a Prema PRO-739 visible spectrophotometer. Statistical significance was determined using one-way ANOVA and Tukey’s *post hoc* tests. All statistical tests were performed using GraphPad Prism version 8.0.

## Supplementary Material

Supplemental file 1
