# Supplementary material for: Posttranscriptional Regulation of tnaA by Protein-RNA Interaction Mediated by Ribosomal Protein L4 in Escherichia coli
Source: J Bacteriol. 2020 Apr 27;202(10):e00799-19. doi: 10.1128/JB.00799-19 (PMC7186457; doi:10.1128/JB.00799-19)
Supplement: Supplemental file 1 [file JB.00799-19-s0001.pdf]

Supplementary figure legends

Fig. S1. The effect of ectopic expression of FLAG-L4 on the stability of TnaA protein after chloramphenicol (Cm) treatment of *E. coli* N3433. (A) Treatment with chloramphenicol to block new protein synthesis and determination of TnaA protein degradation over time by Western blot analysis. After induction with IPTG (0.5 mM) for 30 min at 32°C, 180 rpm, the bacterial culture of the wild-type *E. coli* N3433 strain carrying plasmid pPW500flag (labeled as control) or plasmid pPWflagL4 encoding FLAG-L4 (labeled as L4) was treated with the chloramphenicol. Cells were harvested immediately after stopping translation with chloramphenicol (used as a t = 0 sample) and for the indicated time (2 to 128 minutes). As shown, the same amount of cell mass was analyzed by Western blot (10% SDS-PAGE) using specific antibodies, α-TnaA and α-FLAG to determine the protein degradation of TnaA and FLAG-L4, respectively. (B) The graph represents values quantified for different time points, with 0 minutes as 100% and measured from at least two independent biological repeats. Results are expressed as means ±SEM.

Fig. S2. Determination of the half-life of tna mRNA of N3433 grown at 37 °C (with or without ectopic expression of L4), or N3431 (*rne*^{ts}) and N3433 (wt) grown at 32 °C or 44 °C. N3433 (*rne*$^{+}$) and N3431 (*rne*^{ts}) are isogenic strains and were used in this study. The methods used for RNA preparation and half-life determination are described in Fig. 2C. As shown at the bottom of each sample (see Fig. 2C-E), the amount of 16S rRNA was used as a sample loading control. The rifampicin was used to block new

synthesis of RNA transcripts. The ratio of the 16S rRNA normalized signal intensity to the 0.25 min time point (as the 100% RNA start time point) was used to determine the percentage of transcripts retained over time, and then the percentage of residual or remaining RNA after rifampicin over time was plotted (on the $\log_{10}$), as shown. RNA half-lives were determined by non-linear regression curve fitting (one phase decay) using GraphPad Prism. (A) The half-life of *tna* transcript in N3433 with or without L4 ectopic expression shown in Fig. 2C. (B-C) The half-life of *tna* transcripts in N3431 *rne*^{ts} strain (B) and N3433 isogenic strain (C) under permissive (32°C) or non-permissive (44°C) growth temperatures. The graph represents values quantified from two independent biological repeats. Results are expressed as means ±SD. The half-life of each transcript under the specific conditions of two biological replicates is listed in Table S1.

Fig. S3. Similar levels of DHFR were detected in the SDS-denatured (15%) acrylamide gel blot of the DHFR *in vitro* transcription/translation system in the absence of FLAG-L4 or in the presence of the highest amount of FLAG-L4 (109 µM). (Aa) Schematic representation of a synthetic *tna* operon (top; labeled as 1) derived from the DHFR control plasmid (bottom; labeled as 2) for *in vitro* transcription and translation with the PURExpress *in vitro* protein synthesis kit (New England Biolabs, UK). The upstream T7 promoter (P), ribosome binding site (RBS), *Nde*I and *Bam*HI cloning sites, and the T7 terminator (TERM) downstream of the stop codon are indicated, respectively. (Ab) Western blot analysis of TnaA protein produced from the synthetic *tna* operon (lane 1) compared with the negative control plasmid (DHFR, lane 2) after detection by TnaA polyclonal antibody (α-TnaA) on 10% SDS-PAGE. The faint TnaA

band in lane 2 is from a non-specific binding by using of polyclonal α-TnaA. (Ba-b) Amido black stained membrane (Ba) and quantitative DHFR proteins (Bb) from lane 2 (Ba; no L4, DHFR 100%) and 3 (Ba; with L4, DHFR 97%) are shown. Lane 1 (Ba) shows the *in vitro* translation components without any plasmid DNA template (shown by “-”). M stands for protein molecular weight marker. FLAG-L4 concentration was 109 μM.

Fig. S4. Quantitative data for TnaA, L4 and enolase (Eno) expression levels shown in the Western blot of Figure 7A. Western blots of endogenous TnaA, L4, and Eno were detected from cultured cells at the indicated t2 (OD_{600} = ~0.40) to t5 (OD_{600} = ~1.1) growth phase. The integrated band density of individual proteins (y axis) obtained from the Western blots signals for antibody detection against TnaA, L4 or Eno was plotted for each time point (x axis; t2-t5) as shown.

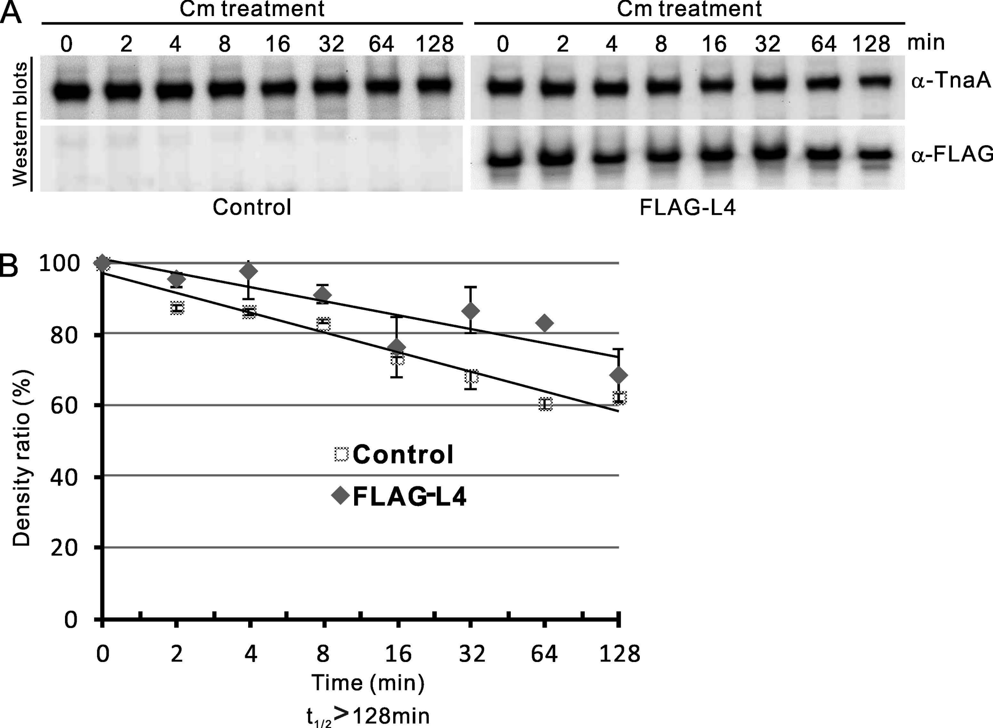
A
Cm treatment
0
2
4
8
16
32
64
128
min
Western blots
α-TnaA
α-FLAG
Control
FLAG-L4
B
100
80
60
40
20
0
Density ratio (%)
Control
FLAG-L4
Time (min)
$t_{1/2}$>128min

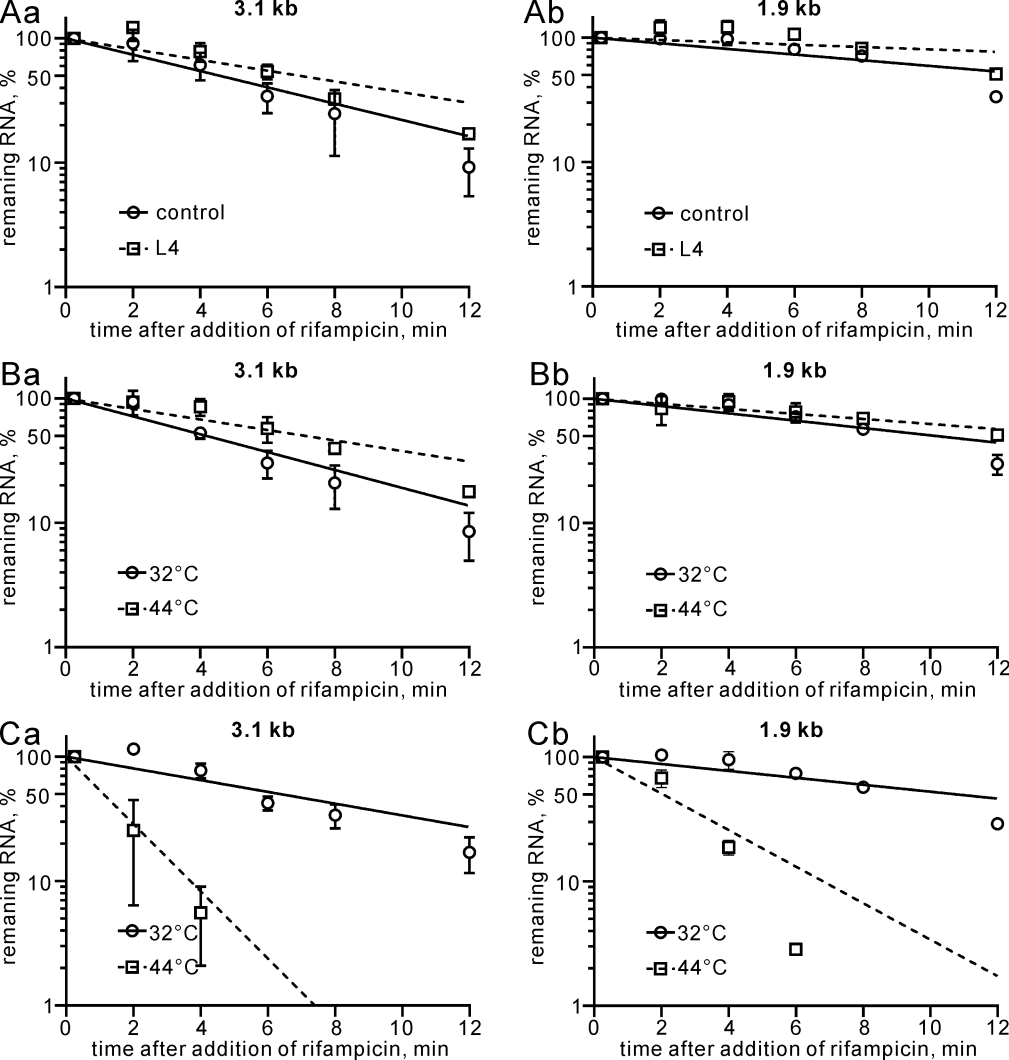
Aa
3.1 kb
Ab
1.9 kb
remaning RNA, %
time after addition of rifampicin, min
control
L4
Ba
3.1 kb
Bb
1.9 kb
32°C
44°C
Ca
3.1 kb
Cb
1.9 kb
100
50
10
1
0
2
4
6
8
10
12

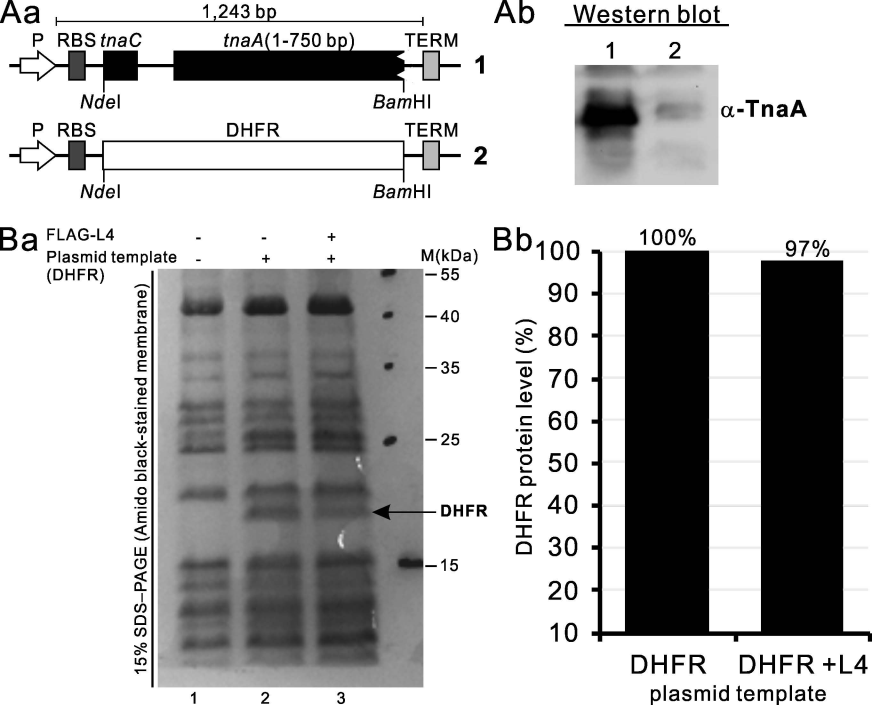
Aa
1,243 bp
P
RBS
tnaC
tnaA(1-750 bp)
TERM
1
NdeI
BamHI
P
RBS
DHFR
TERM
2
NdeI
BamHI
Ab
Western blot
1
2
α-TnaA
Ba
FLAG-L4
-
-
+
Plasmid template (DHFR)
-
+
+
M(kDa)
55
40
35
25
15
DHFR
15% SDS–PAGE (Amido black-stained membrane)
1
2
3
Bb
100%
97%
DHFR protein level (%)
100
90
80
70
60
50
40
30
20
10
DHFR
DHFR +L4
plasmid template

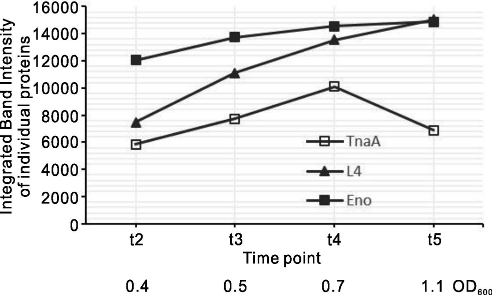
Integrated Band Intensity of individual proteins
16000
14000
12000
10000
8000
6000
4000
2000
0
TnaA
L4
Eno
t2
t3
t4
t5
Time point
0.4
0.5
0.7
1.1
OD_{600}

Table S1. The half-life of two biological replicates (1 and 2) of each *tna* transcript under the specific conditions shown in Fig. 2C and Fig. S2

Experiment		$t_{1/2}$ (min) 3.1 knt (1)	(2)	Average $t_{1/2}$ (min)	SEM (±)	$t_{1/2}$ (min) 1.9 knt (1)	(2)	Average $t_{1/2}$ (min)	SEM (±)
N3433	control	5.9	5.1	5.5	0.4	12.5	14.0	13.3	0.7
	L4	6.6	10.5	8.6	2.0	>13.0	>13.0	>13.0	NA
N3431	32°C	4.4	4.0	4.2	0.2	11.4	9.2	10.3	1.1
	44°C	6.3	8.1	7.2	0.9	>12.0	>12.0	>12.0	NA
N3433	32°C	7.2	5.7	6.4	0.8	12.1	9.1	10.9	1.3
	44°C	<2	<2	<2	NA	<2	<2	<2	NA

Table S2. **List of primers used in this study.**

No.	Primer Name	5′→3′	Use
1.	tnaC-F	TTACGAAGCCGCATTCTGACTGTC	Anti-sense RNA probe 'C'
	tanC-R	TAATACGACTCACTATAGGGGCTATACCGATTAATTCGCC	
2.	tnaA-F	AGATGCGATGGTGCCGATG	Anti-sense RNA probe 'A'
	tnaA-R	TAATACGACTCACTATAGGGACGCAATACTTTCGGTTCG	
3.	tnaB-F	GTCGGCTCCAGTTTTAACACCATC	Anti-sense RNA probe 'B'
	tnaB-R	TAATACGACTCACTATAGGGGCAACAGCTAAGTTAGAGAAC	
4.	TRC-F	CGCGGATCCTTCTTACGTAATTTA	*tna* transcriptional fusion in pMU575
	TRC-R	CCCAAGCTTGAGGCTACATTATTAC	
5.	TSL-F	CCCAAGCTTTTCTTACGTAATTTA	*tna* translational fusion of the *tna* promoter without *tnaC-tnaA* spacer in pMU2386
	TSL-R	CGCGGATCCCCTAAGATATTCATAATGC	
6.	TSL-F	CCCAAGCTTTTCTTACGTAATTTA	*tna* translational fusion of the *tna* promoter with *tnaC-tnaA* spacer in pMU2386
	TSL-spacer-R	CGCGGATCCCCCATTACATAATCCTTC	
7.	LacZ-Rseq	ACTCCAGCCAGCTTTCCG	Sequencing primer for pMU575 & pMU2386
8.	IG-F	TAATACGACTCACTATAGGGTTGCCCTTCTGTAGCCATC	Generating DNA template for *in vitro* transcription of *tnaC-tnaA* spacer (Mobility gel shift assay)
	Ig-R	GAACGGTTCAGGGAGATGTTTAAAGTTTTC	
9.	rpsJ-F	TAATACGACTCACTATAGGGGGCTTGTCGTAGTTGAC	Generating DNA template for *in vitro* transcription of *rpsJ* (*S10-UTR*)
	rpsJ-R	GAGACCAGAGCTCCAATT	
10.	rpsO-F	TAATACGACTCACTATAGGGGCTTAACGTCGCGTAAATTG	Generating DNA template for *in vitro* transcription of *rpsO*
	rpsO R	CTCAGAAACGATTTTAGCTGTTGC	
11.	23S-F	TAATACGACTCACTATAGGGGCCCAGAGCCTGAATC	Generating DNA template for *in vitro* transcription of 23S *rRNA DI*
	23S-R	AGCCTTGGAGGATGGTC	
12.	tna-F	GGAATTCCATATGAATATCTTACATATATGTGTG	Cloning the *tna* DNA template into the DHFR control plasmid
	tnaIVT-R	GCGGGATCCTTAGATGGTCCAGTCTTTGTAT	
13.	tnaR-F	TAATACGACTCACTATAGGGGATTAGATTCAATGTGATC	Generating DNA template for *in vitro* transcription (RNA structural probing)
	tnaR-R	TACATAATCCTTCATTTATTTTAATTACAGTGATCCC	
13.	tnadel-F	P-AATTAAAATAAATGAAGGATTATGTAATGGAAAACT	Generating DNA template for *in vitro* transcription (deletion of *tnaCA* spacer)
	tnadel-R	P-CTACAGAAGGGCAAATCAAGGGC	
13.	tnadel5'-F	P-TCTCGTTTATTTACTTGTTTTAGTAAATGATGG	Generating DNA template for *in vitro* transcription (deletion of 5'-half of *tnaCA* spacer)
	tnadel5'-R	P-TCAAGGGCGGTGATCGACAATTTTG	
13.	tnadel3'-F	P-AGGATTATGTAATGGAAAACTTTAAACATCTC	Generating DNA template for *in vitro* transcription (deletion of 3'-half of *tnaCA* spacer)
	tnadel3'-R	P-AGATGACCTTTGCAAAAGGCAAAATTAAG	

Note: The T7 promoter sequence (TAATACGACTCACTATAGGG) and restriction sites for cloning, *Bam*HI (GGATCC), *Hin*dIII (AAGCTT) and *Nde*I (CATATG), as well as the in-frame (CC) sequences for translation are underlined. F and R represent forward and reverse primers, respectively; P-phospate.
